# Environmentally-Friendly Synthesis of Carbonate-Type Macrodiols and Preparation of Transparent Self-Healable Thermoplastic Polyurethanes

**DOI:** 10.3390/polym9120663

**Published:** 2017-11-30

**Authors:** Seon-Mi Kim, Seul-A Park, Sung Yeon Hwang, Eun Seon Kim, Jonggeon Jegal, Changgyu Im, Hyeonyeol Jeon, Dongyeop X. Oh, Jeyoung Park

**Affiliations:** 1 Research Center for Bio-Based Chemistry, Korea Research Institute of Chemical Technology (KRICT), Ulsan 44429, Korea; atom@krict.re.kr (S.-M.K.); seula@krict.re.kr (S.-A.P.); crew75@krict.re.kr (S.Y.H.); dmstjs0829@naver.com (E.S.K.); jggegal@krict.re.kr (J.J.); 2 Advanced Materials and Chemical Engineering, University of Science and Technology (UST), Daejeon 34113, Korea; 3 Department of Chemical Engineering, Hanyang University, Ansan 15588, Korea; cg.im@sk.com

**Keywords:** thermoplastic polyurethanes, carbonate-type macrodiols, polycondensation, environmentally-friendly synthesis, self-healing elastomers

## Abstract

Carbonate-type macrodiols synthesized by base-catalyzed polycondensation of co-diols and dimethyl carbonate as an environmentally-friendly route were subsequently utilized for the preparation of transparent and self-healable thermoplastic polyurethanes (TPUs) containing a carbonate-type soft segment. Three types of macrodiols, obtained from mono, dual and triple diol-monomers for target molecular weights of 1 and 1.5 kg mol^−1^, were analyzed by ^1^H NMR integration and the OH titration value. Colorless transparent macrodiols in a liquid state at a room temperature of 20 °C were obtained, except the macrodiol from mono 1,6-hexanediol. Before TPU synthesis, macrodiols require pH neutralization to prevent gelation. TPUs synthesized by a solution pre-polymer method with 4,4′-methylene(bisphenyl isocyanate) and 1,4-butanediol as a chain extender exhibited moderate molecular weights, good transparencies and robust mechanical properties. Especially, the incorporation of 3-methyl-1,5-pentanediol within carbonate-type macrodiols enhanced the transparency of the resultant TPUs by decreasing the degree of microphase separation evidenced by ATR-FTIR and DSC. Interestingly, packing density of hard segments and the degree of microphase separation determined the self-healing efficiency of TPUs, which showed good performances in the case of sourced macrodiols from triple diol-monomers.

## 1. Introduction

Demand for the environmentally-friendly production of polyurethanes has attracted much attention because their products are widely used for automotive, houseware, clothing, biomedical products, etc., used by humans [1,2,3]. Recently, green approaches have been developed as follows: (1) polyols have been synthesized from eco-friendly non-petroleum monomers, such as dimethyl carbonate (DMC), instead of toxic phosgene or diphenyl carbonate (DPC) [4,5,6], or renewable feedstock, such as biomass products and carbon dioxide [7,8,9,10,11,12], (2) alternatives to toxic isocyanates or heavy metal catalysts [10,11,12,13,14,15,16], (3) relaxation of manufacturing processes, i.e., from organic solvents to water-based systems or production energy-/time-saving methods [16,17,18], and (4) prolonged lifetime of materials by self-healing [19,20].

Macrodiols used for synthesizing thermoplastic polyurethanes (TPU) are conventionally ether- and ester-types [21,22]. To improve flexibility, hydrolytic stability and abrasion resistivity, carbonate-type macrodiols can be a solution to the problem [4,5,6,18,23,24,25,26,27,28,29,30,31,32,33,34]. Considerable attention has been devoted toward understanding the chemical structure-property relationships of carbonate-type macrodiols and subsequent TPUs [5,6,18,23,24,25,26,27,28,29]. Moreover, important characteristics of carbonate-type macrodiols and subsequent TPUs, such as biocompatibility [30,31], transparency [32,33] and self-healing [34,35], were introduced. However, most researchers have used commercially available macrodiols [24,25,26,27,28,29,30,31,32,33,34].

Bulk production processes of polycarbonates typically use phosgene or DPC for generating carbonate linkages [36,37,38]. For aliphatic carbonate-type macrodiols, DMC can be a greener alternative because it does not generate acids or phenols. DMC is more cost-effective because it is the precursor for DPC production [37]. However, the presently commercialized process for carbonate-type macrodiols needs an elevated pressure to capture volatile DMC at high temperatures to reduce production time, as well as toxic heavy-metal catalysts for transesterification reactions [4,5,39]. On the other hand, the transesterification of cyclic carbonates with diols is accompanied by decarboxylation, which produces unpleasant ether-carbonate-types [23].

Recently, smart approaches to overcome low DMC activity for the synthesis of polycarbonates [40,41] have been reported. Li et al. developed a TiO_2_/SiO_2_/poly(vinylpyrrolidone)-based catalyst suitable for polycondensation with aliphatic diols to obtain high molecular weights (*M*_w_ up to 166,000 g mol^−1^) [42,43]. Lee et al. reported synthesis of high-molecular-weight aliphatic polycarbonates (*M*_w_ up to 200,000 g mol^−1^) catalyzed by simple alkali metal bases, followed by chopping into macrodiols and polyols useful for the polyurethane industry [44,45,46,47]. However, polyurethane synthesis was not experimentally verified. Moreover, the direct preparation of macrodiols without using high-*M*_w_ polymers would reduce production costs.

Herein, we report the straightforward preparation of carbonate-type macrodiols by the polycondensation of DMC with co-diols catalyzed by alkaline “green” catalysts. The feed ratio of DMC to diols was precisely controlled to synthesize target molecular weights, confirmed by both nuclear magnetic resonance (NMR) analysis and the OH titration value. Interestingly, the pH adjustment of macrodiols with a mild acid solution was required to further the TPU preparation. Macrodiols from dual and triple diol-monomers have a good flowability and give subsequent TPUs better transparency, in contrast to the macrodiol obtained from the mono diol in an opaque wax state. Moreover, TPUs synthesized from carbonate-type macrodiols exhibited robust mechanical properties and self-healing features superior to ether-type TPUs. The chemical composition and length of carbonate-type macrodiol-related TPU properties were comprehensively investigated. The transparent self-healable TPU material can be applied as paint protection films in automotive industries.

## 2. Materials and Methods

### 2.1. Materials

1,6-Hexanediol (HD, 97%), 1,4-butandediol (BD, 99%), 3-methyl-1,5-pentanediol (PD, 98%), sodium hydride (NaH, 60% dispersion in mineral oil), dibutyltin dilaurate (DBTDL, 95%), dimethyl carbonate (DMC, 99%), polytetramethylene ether glycol (PTMG, *M*_n_ of 1.0 kg mol^−1^), 4,4′-methylene(bisphenyl isocyanate) (MDI, 98%), potassium hydroxide (KOH, 85%), pyridine (99%), acetic anhydride (99%) and potassium hydrogen phthalate (certified secondary standard reference) were purchased from Sigma Aldrich Corp., St. Louis, MO, USA. *N*,*N*-dimethylacetamide (DMAc, 99%) and tetrahydrofuran (THF, HPLC-grade) were purchased from Alfa Aesar, Ward Hill, MA, USA. All chemicals were used without further purification.

### 2.2. Characterization of OH Titration Value

The hydroxyl (OH) values of macrodiols were determined by titration in accordance with the ASTM method E 222. KOH (28.05 g, 0.500 mol) was dissolved in distilled water (100 mL) and then filled with ethanol to prepare a 1 N KOH solution. A corrected concentration of KOH solution was determined by a titration with potassium hydrogen phthalate. A pyridine-acetic anhydride (500 mL, 3:1, *v*:*v*) solution was prepared. For the blank titration, pyridine-acetic anhydride solution (5.0 mL) was mixed with distilled water (1 mL) and then titrated with 1 N KOH solution. For the sample titration, pyridine-acetic anhydride solution (5.0 mL) was mixed with the sample (1 g) and then stirred at 100 °C for 1 h. Distilled water (1 mL) was added and then stirred at 100 °C for 30 min. After cooling to room temperature of 25 °C, the mixed solution was titrated with 1 N KOH solution. The equations for the OH value and *M*_n_ (titration (titr)) are shown below, where *A* is the consumed volume (mL) of the KOH solution for a blank titration, *B* is the consumed volume (mL) of the KOH solution for the sample titration, *C* is the corrected concentration (N) of the KOH solution and *D* is the used g of sample, respectively.

(1) $$\mathrm{OH}\;\mathrm{value}\;({\mathrm{mg}\;\mathrm{KOH}\;g}^{-1})=\frac{56.11\times \left(A-B\right)\times C}{D}$$

(2) $$M_{n}\;(\mathrm{titr})\;(g{\mathrm{mol}}^{-1})=\frac{56.11\times 1000\times 2\;\left(the\;number\;of\;hydroxyl\;groups\;per\;polymer\;chain\right)}{OH\;value}$$

### 2.3. Brookfield Viscosity, ^1^H NMR, GPC, ATR-FTIR and Transmittance

Viscosities of macrodiols were measured at 25 °C using a Brookfield DV-2 Viscometer (Brookfield Instruments, Middleboro, MA, USA) with an HB-1 spindle at 10 rpm. ^1^H NMR spectroscopic experiments were carried out using a Bruker Avance 300-MHz spectrometer (Billerica, MA, USA). CDCl_3_ was used as the deuterium solvent. Molecular weights and molecular weight distributions of the polymers were measured using gel permeation chromatography (GPC). The experiments were performed using ACQUITY APC XT columns (Waters Corp., Milford, MA, USA) at 40 °C equipped with an ACQUITY refractive index (RI) detector using THF as an eluent. The number-averaged molecular weight (*M*_n_), weight-averaged molecular weight (*M*_w_) and polydispersity index (PDI) were calculated relative to linear polystyrene standards. Attenuated total reflectance–Fourier-transform infrared (ATR-FTIR) spectroscopy was performed using a Nicolet iS50 instrument (Thermo Fisher Scientific, Waltham, MA, USA). All TPU films were scanned 64 times at a resolution of 4 cm^−1^. Transmittance experiments of the films were performed on a UV-2600 (Shimadzu Corp., Kyoto, Japan) UV-Vis spectrometer at a resolution of 0.1 cm^−1^.

### 2.4. DSC and TGA

The glass transition temperature (*T*_g_) was determined by differential scanning calorimetry (DSC) using a Q25 apparatus (TA instruments, New Castle, DE, USA). The measurements were carried out at a heating and cooling rate of 10 °C min^−1^ from −90 °C–90 °C (macrodiols) or 220 °C (TPUs) in a nitrogen atmosphere. The decomposition temperature was determined using a Pyris 1 TGA apparatus (PerkinElmer Inc., Waltham, MA, USA). The samples were heated from 25 °C–800 °C at a heating rate of 10 °C min^−1^ under a nitrogen flow of 50 mL min^−1^. The 5% weight loss temperature was coded as *T*_d5_. The maximum degradation rate temperature was coded as *T*_max_.

### 2.5. Tensile and Self-Healing Tests

Tensile properties were measured using a universal testing machine (UTM) of Instron (Norwood, MA, USA), Model 5943, loaded with a 1-kg load cell in accordance with the ASTM method D 638. The test specimens were cut into dumbbell-shaped bars with dimensions of 63.50 mm × 3.18 mm × 0.3 mm. The test was carried out at a constant speed of 100 mm min^−1^ at 25 °C. Scratch recovery tests were performed by scratching neat TPU films with an 18 G needle [19]. Scratches were crossed over each other obliquely to easily track the changes of the scratch-width using an optical microscope of Axio Imager.A2m (Carl ZEISS, Oberkochen, Germany) equipped with a heating stage. The healing efficiency (%) is defined as 100 × [1 − (final width)/(initial width)] and cited as the average value of five experiments.

### 2.6. Synthesis of Carbonate-Type Macrodiols by Base-Catalyzed Polycondensation of DMC and Co-Diols

A typical synthetic procedure of **M3-1K** (target *M*_n_ of 1 kg mol^−1^ from triple diol-monomers, molar feed ratio of HD:BD:PD = 2:2:1) is described below. HD (18.91 g, 160 mmol), BD (14.42 g, 160 mmol), PD (9.45 g, 80 mmol), DMC (41.44 g, 460 mmol, [DMC]:[co-diols] = 1.15:1) and NaH (0.0320 g, 0.80 mmol, 0.2 mol % to co-diols) were added into a 500-mL round-bottomed flask equipped with a distillation apparatus. The reaction temperature was increased to 120 °C and maintained for 2 h in nitrogen atmosphere. The generated transesterification byproduct, methanol, was gradually distilled off. The distillation pressure was steadily reduced to 10 Torr for 6 h, while the temperature was gradually increased to 190 °C. After the pressure was returned to atmospheric pressure by a nitrogen purge followed by cooling to room temperature of 25 °C, the reactant dissolved in dichloromethane (300 mL) was neutralized with hydrochloric acid solution (0.1 M), then washed with water to remove residual acids and salts. The solution containing reactant was distilled off and dried under vacuum at 80 °C for 24 h. *M*_n_ (NMR): 1010 g mol^−1^, *M*_n_ (titr): 990 g mol^−1^, ^1^H NMR (300 MHz, CDCl_3_): δ 4.28–4.07, 3.73–3.61, 1.78–1.72, 1.68–1.57, 1.54–1.49, 1.39–1.30, 0.96–0.89.

### 2.7. Synthesis of TPUs by a Solution Pre-Polymer Method

A typical polymerization procedure of **M3-1K-TPU** (molar feed ratio of **M3-1K**:MDI:BD = 1:2:1, content of hard segment = 38 wt %) is described below. **M3-1K** (10 g, 10 mmol) in a dried glass vessel equipped with a mechanical stirrer was heated in an oil bath at 100 °C under vacuum (<133 Pa) for 1 h to remove any moisture and then cooled to 70 °C. Dried MDI (5.25 g, 21 mmol) and DBTDL (0.032 g, 2000 ppm) dissolved in DMAc (15 mL) were added dropwise into the vessel and stirred for 2 h under a nitrogen atmosphere. After the synthesis of the bis-isocyanate-terminated pre-oligomer, the reactor was cooled to room temperature of 25 °C, and BD (0.90 g, 10 mmol) as a chain extender dissolved in DMAc (10 mL) was added to the reactor at 60 °C. The reaction was continued until the NCO peak disappeared in the FT-IR spectrum, which required 1.5 h for **M3-1K-TPU**. The final concentration of the TPU solution was adjusted to 30 wt % by adding additional DMAc (13 mL). *M*_w_ (GPC): 73,600 g mol^−1^, PDI: 2.79, ^1^H NMR (300 MHz, CDCl_3_): δ 7.39–7.20, 7.15–7.01, 4.13, 3.91, 1.87–1.73, 1.72–1.63, 1.54, 1.47–1.31, 0.99.

## 3. Results and Discussion

### 3.1. Carbonate-Type Macrodiols by Base-Catalyzed Polycondensation of DMC and Co-Diols

**Scheme 1:**
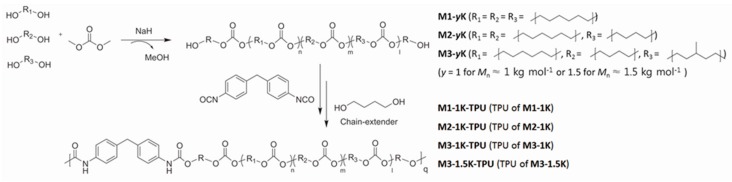
Synthesis of carbonate-type macrodiols and thermoplastic polyurethanes (TPUs).

**Scheme 2:**
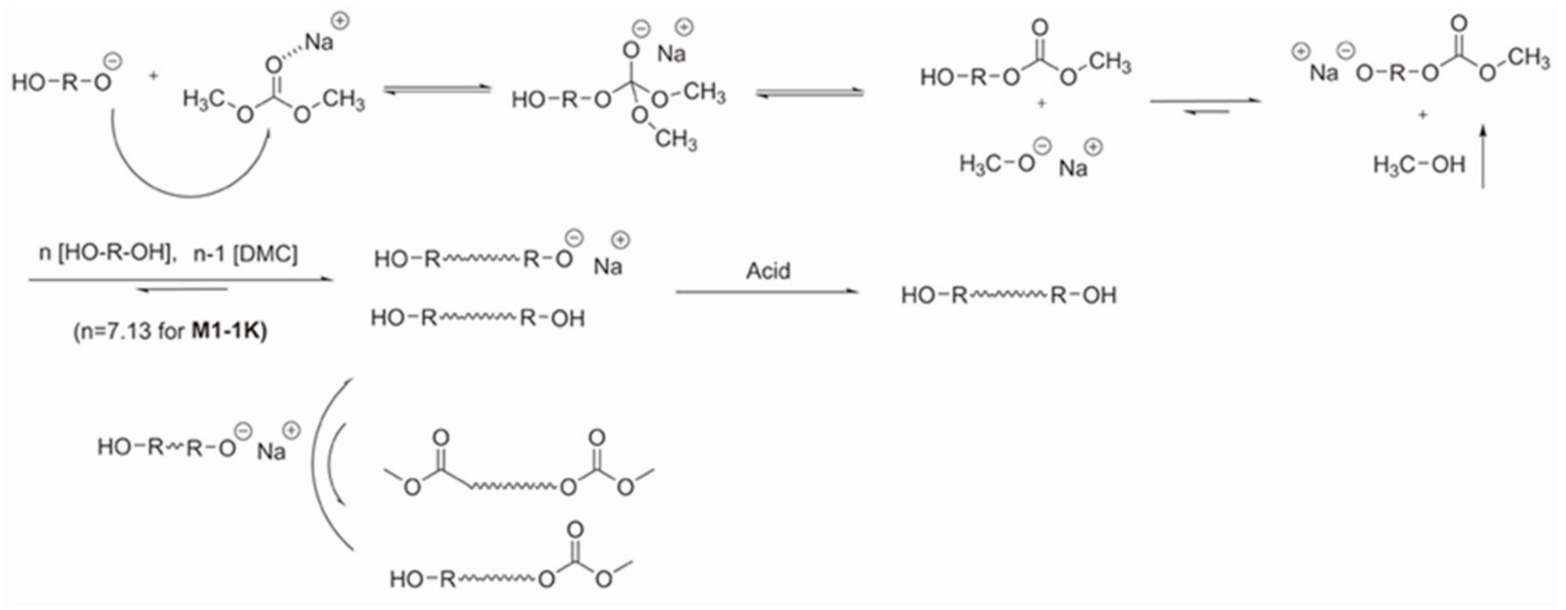
Base-catalyzed polycondensation mechanism of diol and dimethyl carbonate (DMC) for the direct synthesis of macrodiols.

The synthesis route for carbonate-type macrodiols and detailed polycondensation mechanism of diol and DMC for the direct synthesis of macrodiols are shown in Scheme 1 and Scheme 2. Transesterification is activated by the presence of an alkali base, which attacks the carbonyl carbon of the DMC and growing methyl carbonate chain-end, forming a tetrahedral intermediate [44]. A new carbonate linkage is generated by the liberation of a methoxide anion, which is subsequently protonated to be volatile methanol with the re-generation of a new alkoxide anion. The stoichiometric imbalance between diol and DMC considering the evaporated portion leads the step-growth polymers to have bis-hydroxyl-terminated chain-ends at the later stage of the reaction. Possible methyl carbonate chain-ends are statistically attacked by alkoxide anions of the growing bis-hydroxyl chains to liberate methoxide anions, then the methyl carbonate chain-ends transform to hydroxyl chain-ends.

We proposed that the flowability of macrodiols and the properties of subsequent TPUs are both dependent on the composition of co-diols. Three different diol feed compositions of HD, BD and PD were introduced. To compare molecular weight-effects, the molar feed ratio of DMC to total diols was adjusted from 1.10 to 1.20 for the target *M*_n_ of 1 and 1.5 kg mol^−1^, respectively. Little excess molar amount of DMC was fed to diols since the specific portion of volatile DMC was evaporated during the transesterification reaction. When the ratio of DMC to diols exceeded the upper limit, macrodiols contained methyl carbonate chain-ends. In contrast, when the ratio was below the lower limit, target molecular weights of macrodiols were unattainable. The composition of macrodiols was expressed in the form of **M*x*-*y*K**, where *x* and *y* indicate the number of diol-monomers used and *M*_n_ in kg mol^−1^, respectively, e.g., for **M2-1K**, the target *M*_n_ of 1 kg mol^−1^ was from dual diol-monomers (molar feed ratio of HD:BD = 1:1), and for **M3-1.5K**, the target *M*_n_ of 1.5 kg mol^−1^ was from triple diol-monomers (molar feed ratio of HD:BD:PD = 2:2:1), respectively. The detailed synthesis information and molecular weights of macrodiols are shown in Table 1.

**Figure 1:**
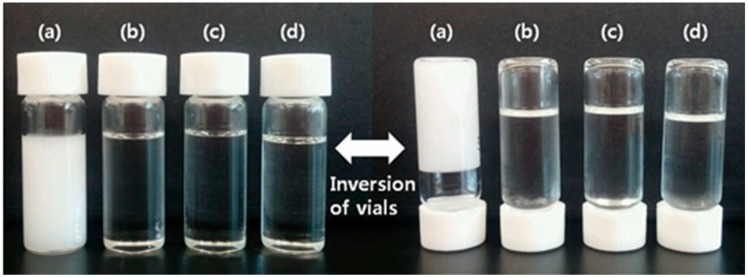
Photographs of the carbonate-type macrodiols of (**a**) **M1-1K**, (**b**) **M2-1K**, (**c**) **M3-1K** and (**d**) **M3-1.5K** at 20 °C.

**Figure 2:**
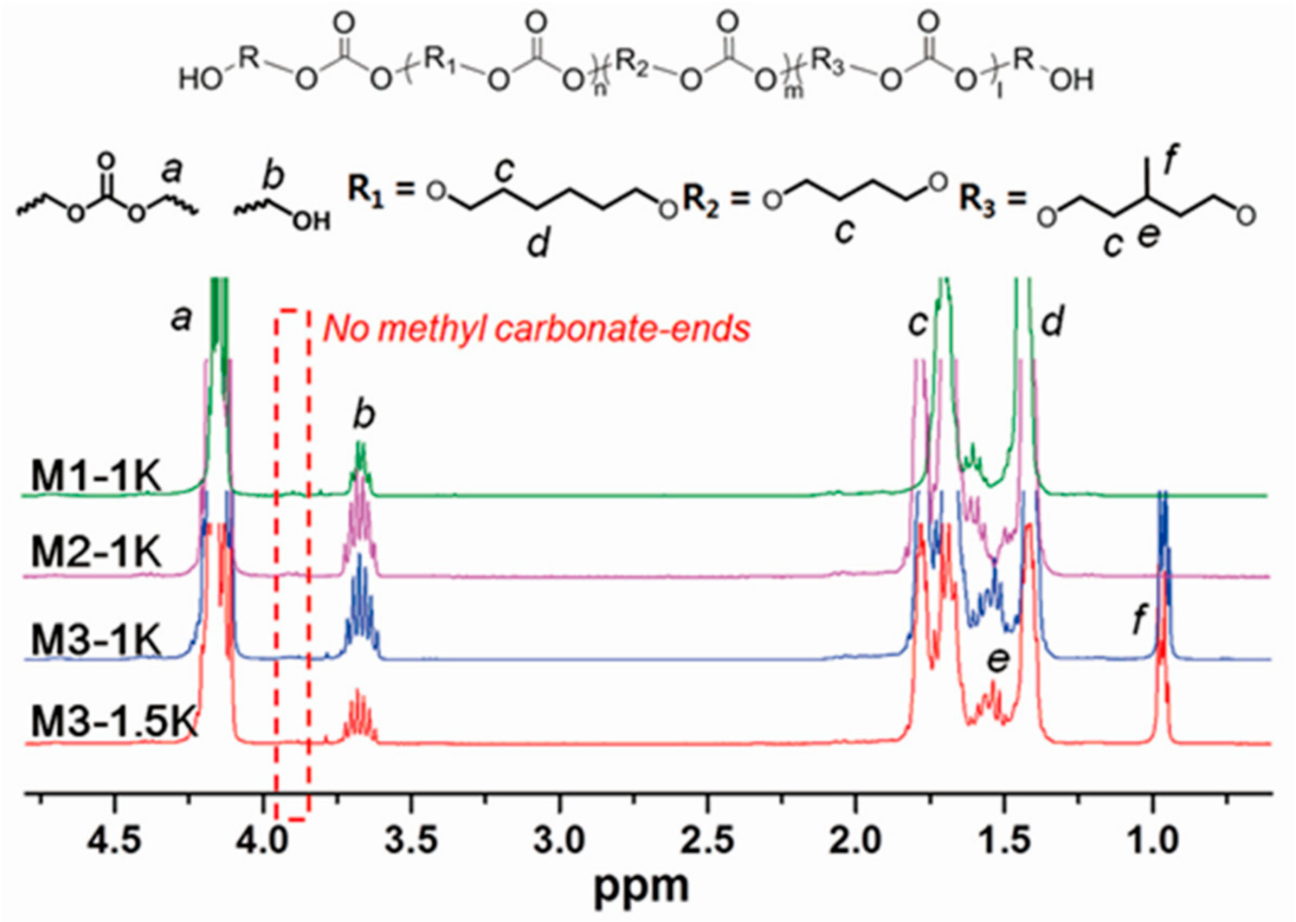
^1^H NMR spectra of carbonate-type macrodiols in CDCl_3_ (300 MHz).

**M1-1K** synthesized by only HD-induced crystallization obtained a solid state (*T*_m_ = 42 °C, Figure S1), whereas other macrodiols synthesized by dual or triple diols showed a good flowability (Figure 1). It should be emphasized that the colors of **M2-1K** and **M3-*y*K** were transparent without yellowness, in sharp contrast to conventional synthesis with DPC and a titanium catalyst that showed a dark brown color (Figure S2). The synthetic procedures for DPC instead of DMC are shown in the Supplementary Materials. The control sample of polytetramethylene ether glycol of *M*_n_ = 1.0 kg mol^−1^ (**PTMG-1K**) showed a wax state (*T*_m_ = 20 °C, Figure S1). Chemical structures and molecular weights of the synthesized carbonate-type polyols were determined by ^1^H NMR spectroscopy (Figure 2). Aliphatic proton peaks of repeating diols appeared at 1.78–0.96 ppm, α-methylene proton peaks (*a*) adjacent to the carbonate group at 4.07–4.28 ppm and α-methylene proton peaks (*b*) adjacent to the end-hydroxyl group at 3.61–3.73 ppm, respectively. There were no traces of DMC and methyl carbonate groups, suggesting that the two chain-ends of oligomers were comprised by only hydroxyl groups. *M*_n_ (NMR) was obtained by calculating the integral of *a* to *b*. The determination of *M*_n_ values should be strictly verified, because following TPU synthesis requires an accurate stoichiometric balance between the macrodiol, diisocyanate and chain extender. The OH value titration was carried out to calculate *M*_n_ (titr), which was closely correlated with *M*_n_ (NMR), suggesting macrodiols were synthesized elaborately. Molecular weights of **PTMG-1K** were also double-checked.

**M1-1K** has a single repeating unit of HD, and **M2-1K** and **M3-1K** consist of multi-repeating units having different domain sizes. **M1-1K** is a crystalline solid due to the effective chain packing of the regular repeating unit, while the others are liquids. Compared to the liquid macrodiols, **M3-1K** exhibited 1.8-fold higher viscosity than that of **M2-1K**. It seems that the incorporation of PD increases the radius of gyration of the macrodiols probably because the methyl pendent group of PD more sterically hinders the conformational chain rotation than the hydrogen pendent group of HD does [48].

### 3.2. Preparation of TPUs Containing a Carbonate-Type Soft Segment

The synthesized macrodiols were subsequently utilized for the preparation of TPUs by a solution pre-polymer method. The macrodiol was reacted with two molar equivalents of diisocyanate monomer (MDI) in a DMAc solvent in the presence of DBTDL as a catalyst to synthesize the bis-isocyanate-terminated pre-oligomer. Then, BD was added to the solution as a chain extender to complete the TPU synthesis. Interestingly, all trials failed because they generated cross-linked gels before the addition of a chain extender, except commercial **PTMG-1K** (Figure S3). However, it was revealed that macrodiols should be treated with an acid solution for neutralization (Scheme 2). Followed by washing with water to remove residual acid and salts, highly viscous TPU solutions were observed. It seems that remaining base alkoxides activate isocyanates and bis-isocyanate-terminated pre-oligomers to form poly(isocyanate)s via anionic polymerization instantly [49]. A plausible cross-linking reaction mechanism of poly(isocyanate)s is shown in Scheme S1.

**Figure 3:**
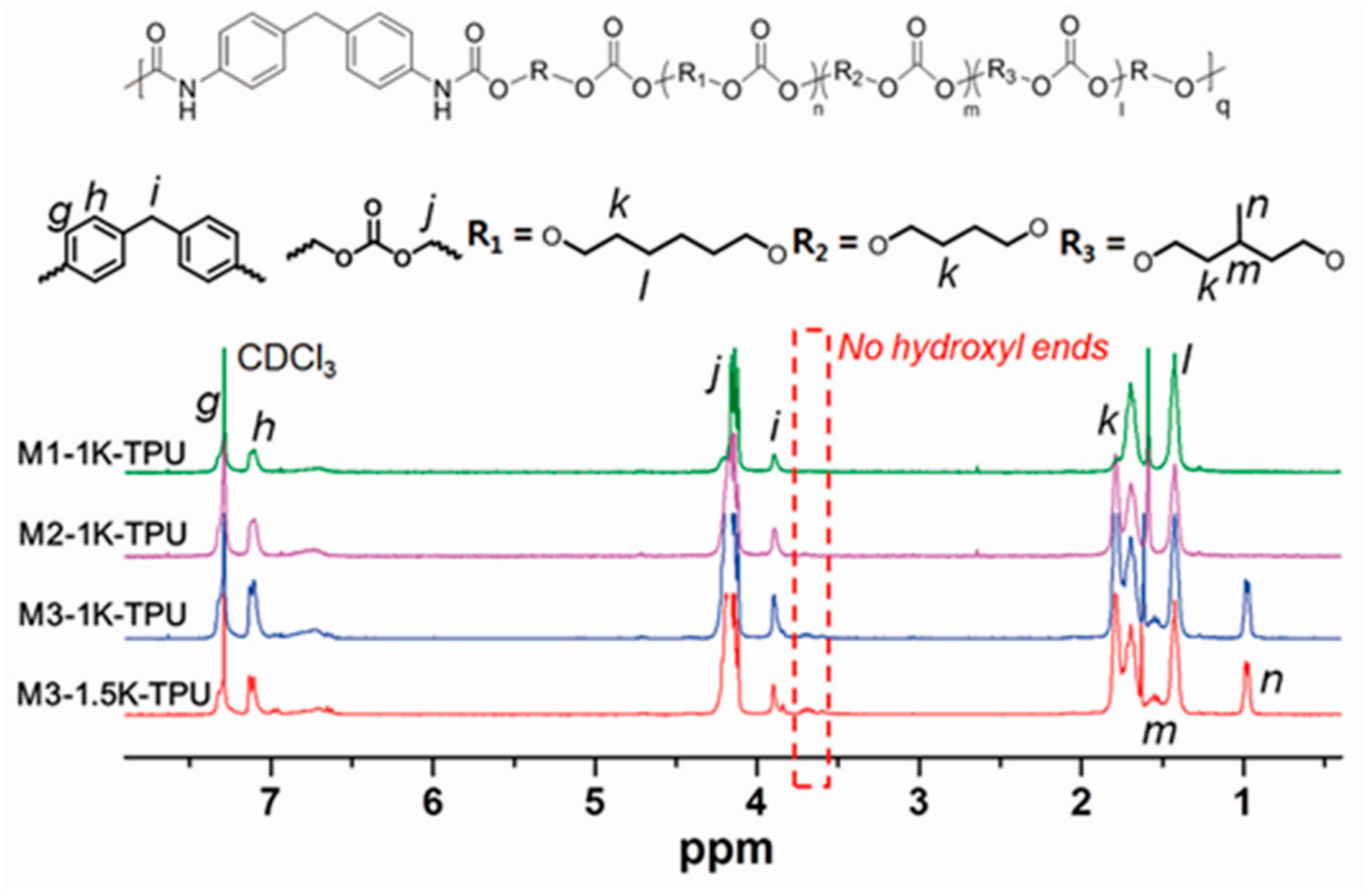
^1^H NMR spectra of TPUs in CDCl_3_ (300 MHz).

Detailed molecular weights and thermal properties of TPUs are shown in Table 2. Chemical structures of TPUs were confirmed by ^1^H NMR spectra (Figure 3). TPUs synthesized from carbonate-type macrodiols showed aromatic and methylene proton peaks of repeating MDI that appeared at 7.39–7.01 and 3.91 ppm, respectively. Proton peaks of repeating macrodiols were observed with well-matched integral ratios compared to two molar equivalents of diisocyanate. There were negligible traces of α-methylene proton peaks adjacent to the macrodiol-end and chain extender at 3.61–3.73 ppm, suggesting the completion of TPU synthesis. The control TPU from ether-type macrodiol (**PTMG-1K-TPU**) was also prepared using the same procedures, which showed a well-assigned ^1^H NMR spectrum (Figure S4).

### 3.3. Transparency of TPUs Related to Microphase Separation

All TPUs showed good solubility in chloroform, THF and DMAc, indicating that all the TPUs are solution-processable and that the chain extender did not cause gelation during polymerization. Molecular weight information was obtained by GPC measurement using THF as a mobile phase (Table 2, Figure S5). The relationship between the highest *M*_w_ of 153 kg mol^−1^ (**M1-1K-TPU**) and the lowest *M*_w_ of 74 kg mol^−1^ (**M3-1K-TPU**) has no tendency; however, *M*_w_ of TPUs over 70 kg mol^−1^ could enable studies of thermal and mechanical properties.

**Figure 4:**
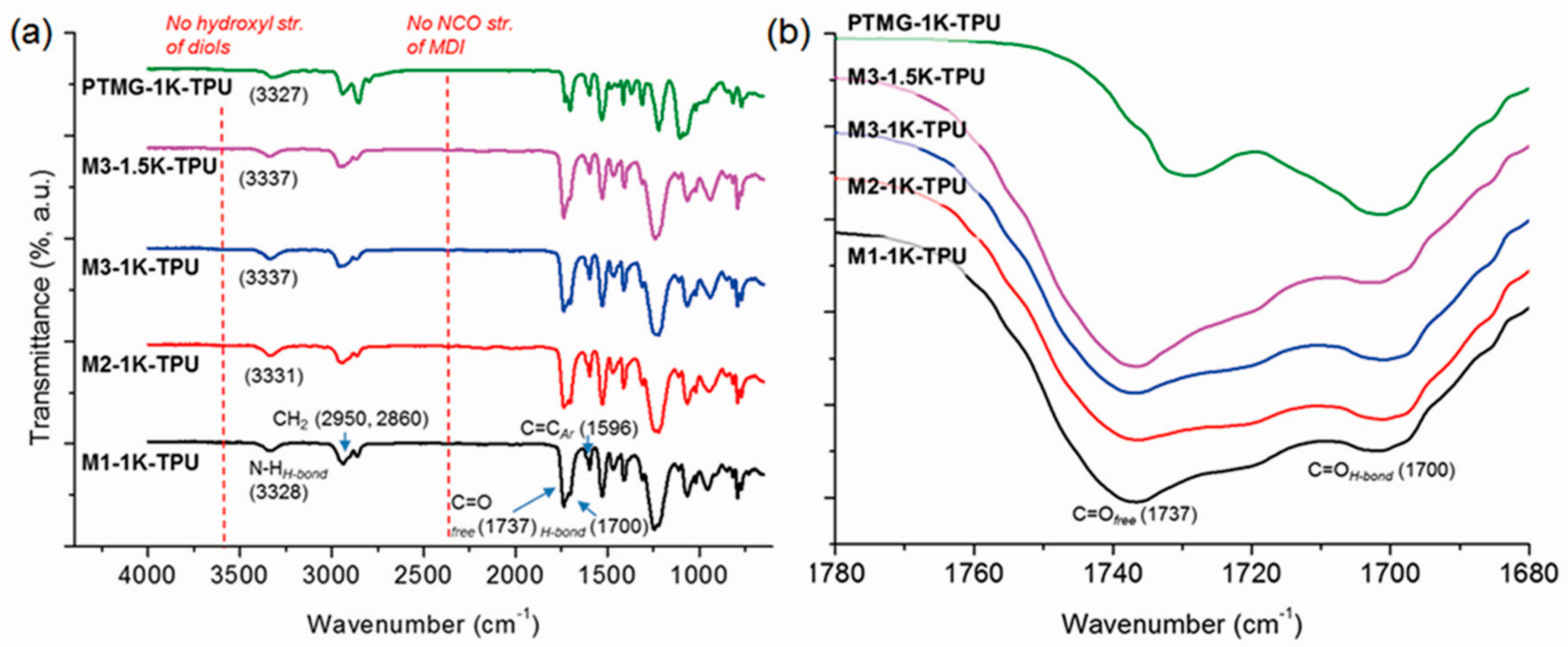
ATR-FTIR spectra of TPUs ((**a**) full range; (**b**) enlarged range to carboxyl stretchings).

Films were prepared from each of the TPUs via solvent casting. A 30 wt % DMAc solution of TPU was loaded into a square mold with dimensions of 70 mm × 50 mm × 10 mm on a Teflon sheet. The mold was gradually heated from 40 °C–130 °C over 24 h. The residual solvent was removed by vacuum drying at 60 °C for 12 h. No bubble was observed inside the samples. Prepared TPU films were characterized by ATR-FTIR spectroscopy (Figure 4). The disappearance of the hydroxyl stretching of macrodiol and chain extender at 3600 cm^−1^ and NCO stretching of MDI at 2300 cm^−1^ indicated the macrodiol, chain extender and diisocyanate monomer were fully converted to urethane bonds [25,27]. All TPUs showed hydrogen bonded N–H stretching around 3327–3337 cm^−1^ without traces of free N–H stretching at 3640 cm^−1^. All the N-H in the hard segment formed hydrogen bonding with carbonyl C=O. The intensity of hydrogen bonding decreased in the following order: **PTMG-1K-TPU** (3327 cm^−1^) > **M1-1K-TPU** (3328 cm^−1^) > **M2-1K-TPU** (3331 cm^−1^) > **M3-*y*K-TPU** (3337 cm^−1^). Carbonate-type soft segments interrupted hydrogen bonding in the hard segments by better miscibility than that of ether-type soft segments, and co-diols enhanced the effectiveness [32]. All TPUs containing carbonate-type soft segments showed both free C=O stretching at 1737 cm^−1^ and hydrogen bonded C=O stretching red-shifted at 1700 cm^−1^ [50]. Unlike TPU containing ether-type soft segments showing larger absorbance at hydrogen bonded stretching, **M1-1K-TPU** showed larger absorbance at free stretching because of carbonate units within soft aliphatic chains. **M2-1K-TPU** and **M3-1K-TPU** (sourced macrodiols from dual and triple diol-monomers) of *M*_n_ ≈ 1 kg mol^−1^ showed a higher absorbance ratio of C=O*_H-bond_*/C=O*_free_*, indicating an increasing portion of aliphatic chains penetrating inside the hard segments. When *M*_n_ of the macrodiol increased to 1.5 kg mol^−1^ (**M3-1.5K-TPU**), the absorbance ratio of C=O*_H-bond_*/C=O*_free_* decreased because of enhanced microphase separation of the soft and hard segments, which is also supported by the following DSC analysis. Other stretching peaks such as CH_2_ anti-symmetry and symmetry of macrodiols and aromatic C=C of MDI appeared at 2950, 2860 and 1596 cm^−1^, respectively.

**Figure 5:**
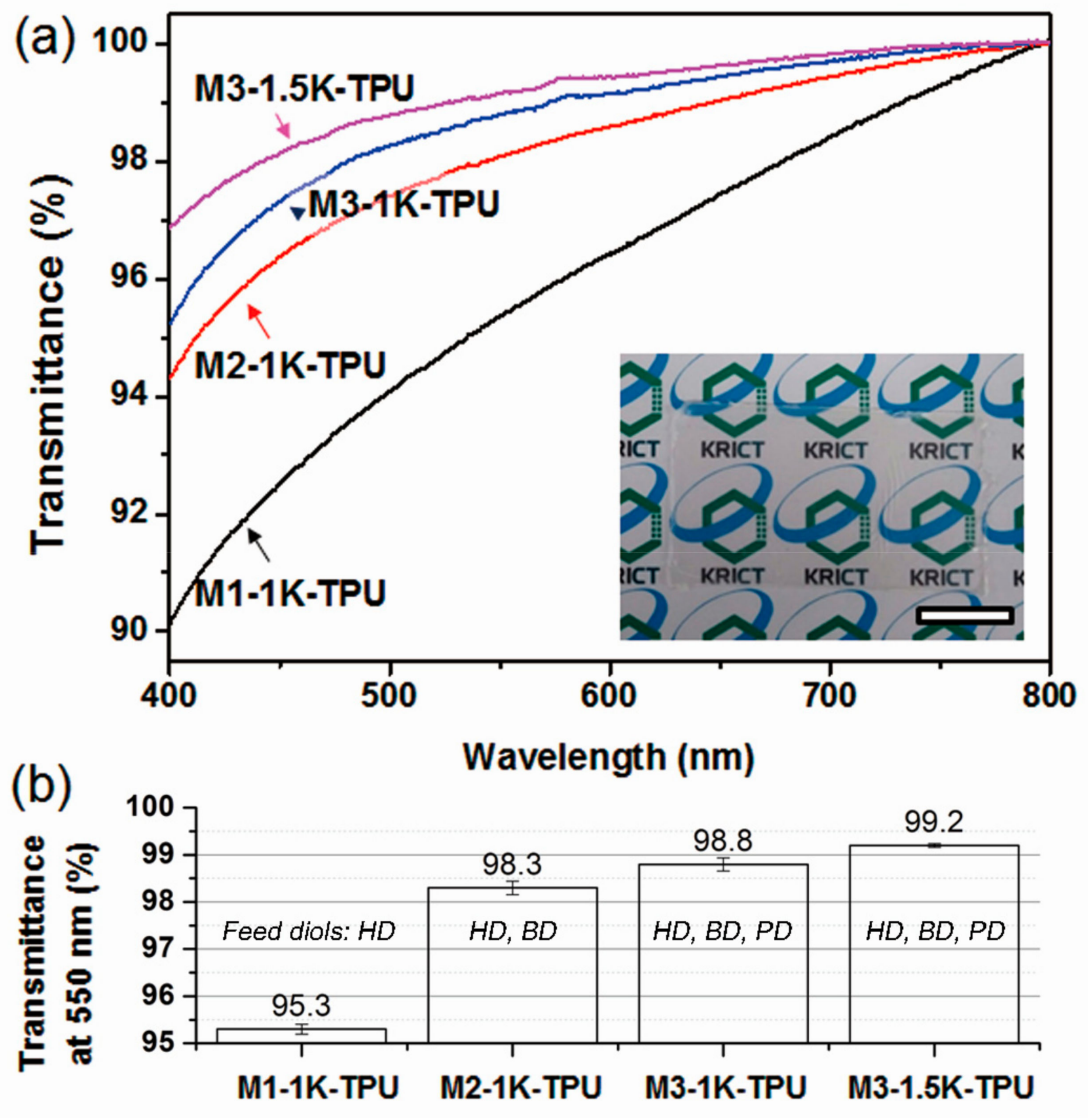
(**a**) Transmission spectra and (**b**) transmittance at 550 nm of TPU films with a thickness of 75 μm (inset: photograph of the film of **M3-1.5K-TPU**; scale bar = 2 cm).

**Figure 6:**
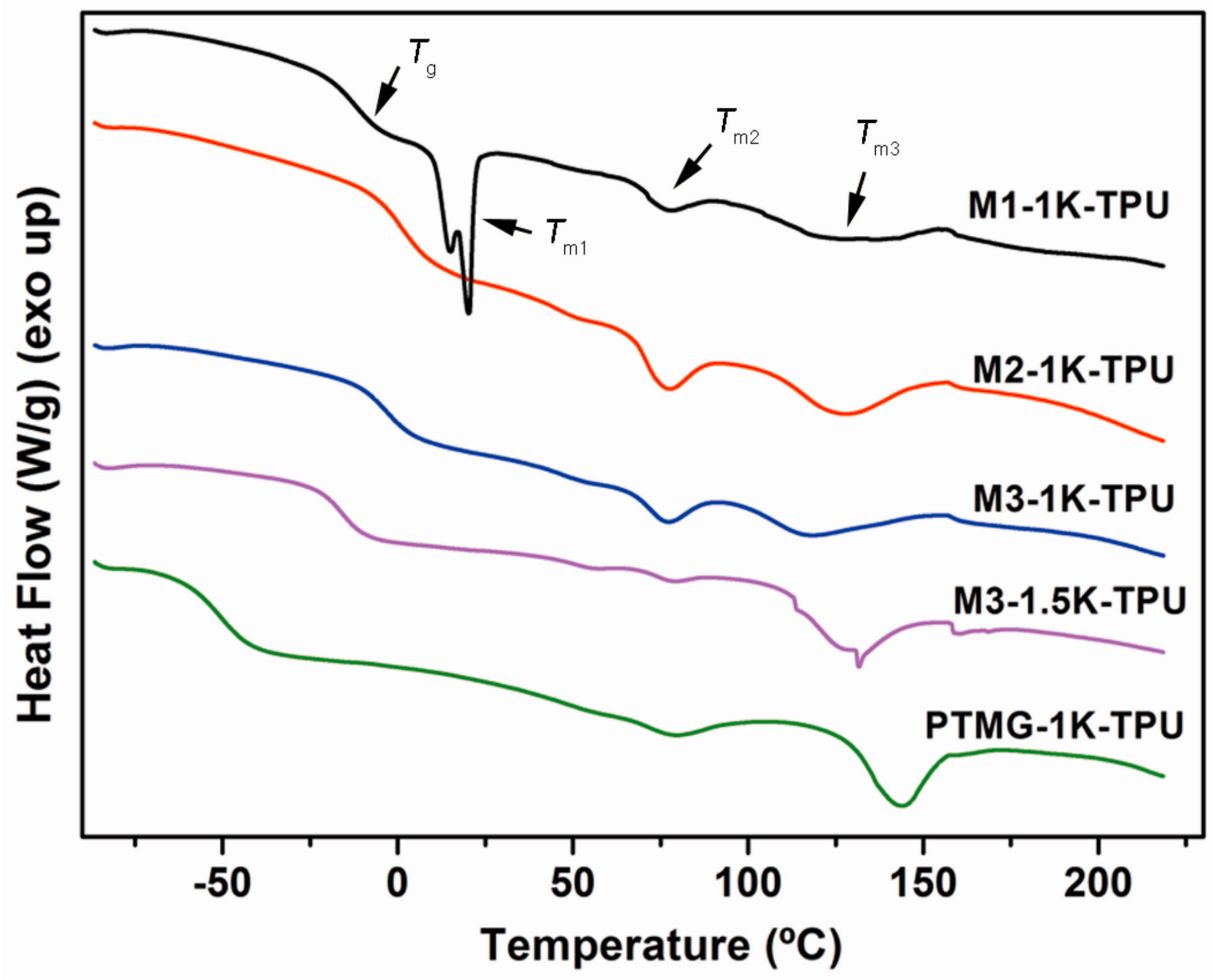
DSC curves of TPUs (first scan).

Films of thickness 75 (±5) µm were subjected to UV-Vis spectroscopy (Figure 5). All samples showed transmittance above 90% at 400–800 nm, and the transparency increased in the order of **M1-1K-TPU** < **M2-1K-TPU** < **M3-1K-TPU** < **M3-1.5K-TPU**. The crystalline soft segments of **M1-1K-TPU** resulted in its lowest transparency (Figure 6). However, the macrodiol from dual diol-monomers increased the transparency of the TPU film significantly. Moreover, macrodiols from triple diol-monomers containing PD were better than those from dual diol-monomers. **M3-1.5K-TPU** containing longer soft chains from the triple diol-monomers exhibited the highest transparency as expected; the transmittance at 550 nm was 99.2 (±0.040)%. Excellent transparency occurred from better miscibility between the co-carbonate-type soft and hard segments [32]. Particularly, the incorporation of PD having a methyl branch within the macrodiols decreased the crystallinity of the hard segment efficiently, as proven by the following DSC analysis [51,52].

### 3.4. Thermal and Mechanical Properties

The *T*_g_s of the synthesized TPUs were measured by DSC (Table 2, Figure 6). Among the TPUs from the carbonate-type macrodiols with *M*_n_ ≈ 1 kg mol^−1^, **M1-1K-TPU** exhibited both *T*_g_ (−12 °C) and *T*_m1_ (20 °C) of the soft segments. The degree of crystallinity of the hard segments can be compared with the melting enthalpy (△*H*_m_). **M3-1K-TPU** exhibited lower △*H*_m_ values than **M2-1K-TPU** did owing to the methyl branch within the macrodiols hindering the crystallization of hard segments. **M3-1.5K-TPU** obtained from the longer macrodiol exhibited a lower *T*_g_ (−16 °C) and △*H*_m2_ (0.5 J g^−1^) and a higher *T*_m3_ (132 °C) and △*H*_m3_ (6.4 J g^−1^) than **M3-1K-TPU** did (*T*_g_ = −2.9 °C, △*H*_m2_ = 1.8 J g^−1^, *T*_m3_ = 117 °C and △*H*_m3_ = 3.9 J g^−1^) since better microphase separation significantly reduced the metastable crystalline phase expressed by the value of △*H*_m2_.

**Figure 7:**
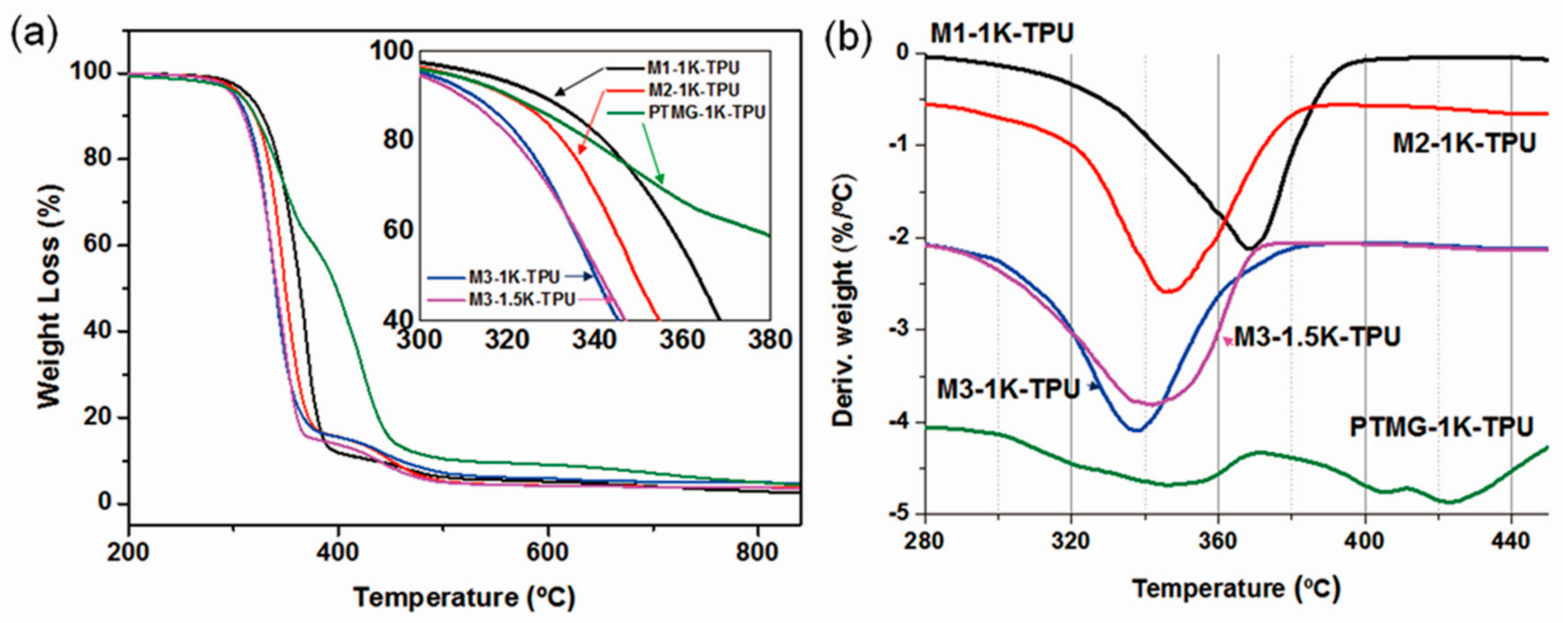
(**a**) TGA and (**b**) DTG curves of TPUs.

*T*_d5_s, indicating the initial thermal stability of TPUs determined by TGA, decreased in the following order (Table 2, Figure 7): **M1-1K-TPU** (314 °C) > **M2-1K-TPU** (306 °C) > **M3-1K-TPU** (301 °C) > **M3-1.5K-TPU** (299 °C). The order of thermal stability of the TPUs can be explained by the bond dissociation energy [53]: the C−C bond is stronger than the C−O bond, and the C–C bond bearing an alkyl substituent instead of hydrogen is weaker [54]. **M1-1K-TPU** containing a lower weight percentage of C−O bond compared to other polymers demonstrated better thermal stability. Further, **M3-*y*K-TPU**s containing methyl substituents were less thermally stable than **M1-1K-TPU** and **M2-1K-TPU** containing the stronger C−C bond. **PTMG-1K-TPU** exhibited two separate peaks in the DTG curve and an unsharp weight-loss in the TGA curve, because crystalline PTMG with lower oxygen contents endures at a higher temperature [55,56,57].

**Figure 8:**
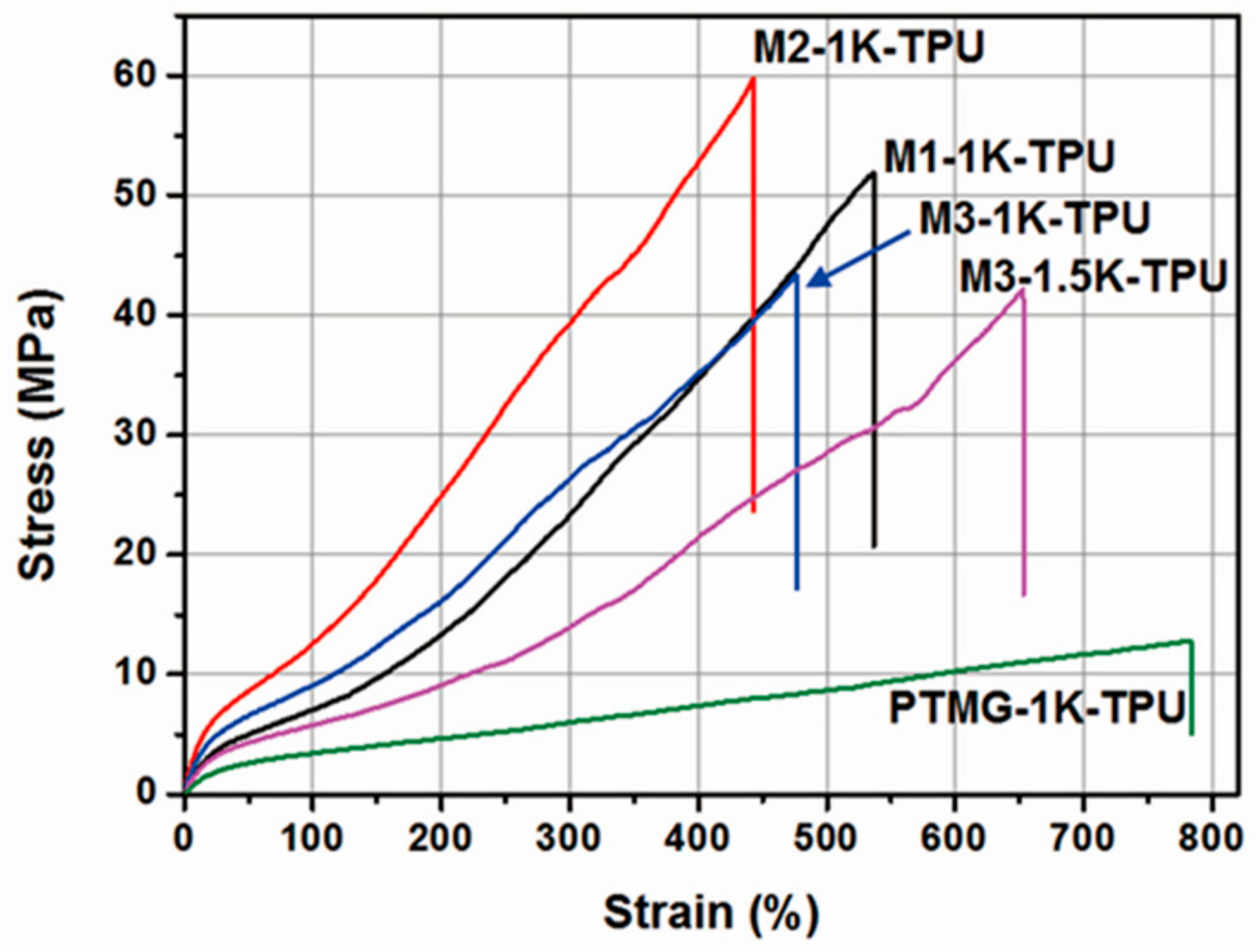
Stress-stain curves of TPU films.

Tensile tests of TPU films were carried out using a UTM obeying an ASTM method (Table 3, Figure 8). Modulus at 100% strain, ultimate tensile strength (UTS) and toughness of TPU films containing carbonate-type soft segments reached over 5.7 MPa, 44 MPa and 100 MJ m^−3^, respectively, which showed superior values to **PTMG-1K-TPU** (3.3 MPa, 9.9 MPa, 40 MJ m^−3^, respectively). Interestingly, **M3-1.5K-TPU** exhibited a robust UTS of 44 MPa compared to that of **M3-1K-TPU** (46 MPa) and increased the elongation at break to 680%, which was higher than 470%. The methyl branch within carbonate-type macrodiols decreased the packing density of hard segments evidenced by ATR-FTIR and DSC investigations, which could possibly explain the slight retreating of modulus and UTS [23,52].

### 3.5. Self-Healing Properties

**Figure 9:**
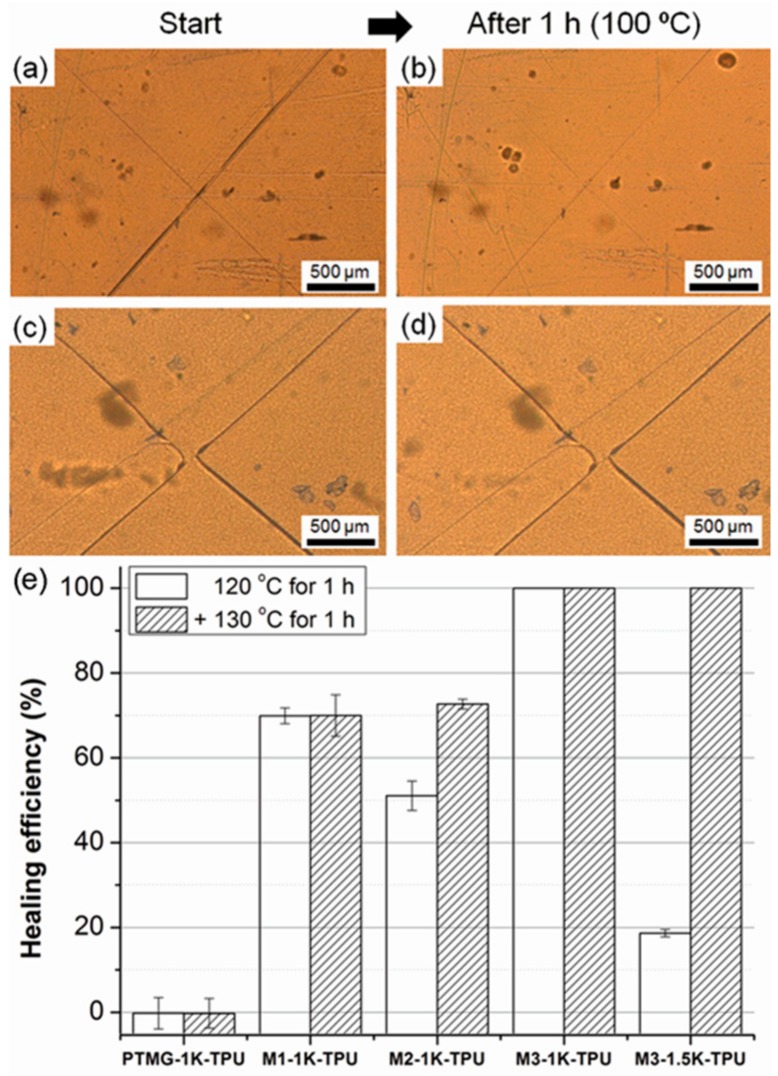
Optical microscopy images of the X-shaped scratch on the TPU films of (**a**,**b**) **M3-1K-TPU** and (**c**,**d**) **PTMG-1K-TPU** before and after healing for 1 h at 100 °C. (**e**) Healing efficiencies of TPU films after 1 h at 120 °C and +1 h at 130 °C.

It is expected that the decreased packing density of hard segments induces intrinsic self-healing, because relatively poor hydrogen bonding assists the diffusion of polymer chains [19]. The self-healing properties were evaluated by a scratch recovery test of TPU films [58,59]. A 20–60 μm-wide scratch was placed on a heating stage, and the scratch width was monitored using optical microscopy. The microscopic images are shown in Figure S6. Healing efficiencies of **M3-1K-TPU** were the best with 72 (±2.0)% for 1 h at 100 °C and 100% for 1 h at 120 °C, whereas **PTMG-1K-TPU** showed poor efficiencies (Figure 9). The healing efficiencies of **M1-1K-TPU** and **M2-1K-TPU** were less than that of **M3-1K-TPU**. This suggests that relatively insufficient crystallinity of hard segments increased the mobility of polymer chains at a given temperature condition. Healing efficiencies of **M3-1.5K-TPU** from the longer macrodiol were short of **M3-1K-TPU** at 120 °C because better efficient microphase separation hindered the diffusion of polymer chains.

## 4. Conclusions

Three different compositions of diols selected from HD, BD and PD were reacted with DMC by environmentally-friendly base-catalyzed polycondensation to synthesize four types of carbonate-type macrodiols. Macrodiols synthesized from dual or triple diol-monomers exhibited a good flowability and transparency, except a macrodiol from only HD (**M1-1K**). After the neutralization of macrodiols, TPUs could be prepared by a solution pre-polymer method. NMR, GPC and ATR-FTIR data indicated successful molecular weights >70 kg mol^−1^ without a trace of monomers and decreased intensity of hydrogen bonding of the hard segment as the number of monomers increased. Toughness values of all TPUs containing carbonate-type soft segments more than doubled that containing ether-type soft segments, and comparable thermal stabilities were observed. The methyl branch of macrodiols was fundamental for the high transparency and self-healing properties of TPU films by better miscibility of the soft and hard segment interrupting the crystallinity. This DMC-mediated macrodiol and subsequent approach to TPUs is expected to suggest eco-friendly production in the polyurethane industry.

## Figures and Tables

**Table 1:** Polymerization information and characterizations of carbonate-type macrodiols. HD, 1,6-hexanediol; BD, 1,4-butandediol; PD, -methyl-1,5-pentanediol; PTMG, polytetramethylene ether glycol.

Sample	Feed Ratio ^1^ (HD:BD:PD)	[DMC]/[co-diols] ^2^	*M*_n_ (NMR) ^3^ (g mol^−1^)	OH Value (mg KOH g^−1^)	*M*_n_ (titr) ^4^ (g mol^−1^)	State ^5^	Viscosity ^6^ (cP)
**M1-1K**	1:0:0	1.10	1180	104	1080	Wax	-
**M2-1K**	1:1:0	1.15	940	122	920	Clear oil	3700
**M3-1K**	2:2:1	1.15	1010	113	990	Clear oil	6800
**M3-1.5K**	2:2:1	1.20	1720	70	1610	Clear oil	12,200
**PTMG-1K**	-	-	1000	117	960	Wax	-

^1^ Based on the initial molar ratio of HD, BD and PD; ^2^ based on the initial molar feed ratio of DMC to co-diols; ^3^ determined by ^1^H NMR analysis; ^4^ calculated from the OH value; ^5^ observed state of macrodiols at 20 °C; ^6^ measured by the Brookfield DV2 Viscometer at 25 °C.

**Table 2:** Thermal characterizations of TPU.

Sample	*M*_w_^3^ (g mol^−1^)	PDI	*T*_g_^4^ (°C)	*T*_m2_ (°C)	△*H*_m2_ (J g^−1^)	*T*_m3_ (°C)	△*H*_m3_ (J g^−1^)	*T*_d5_^5^ (°C)	*T*_max_ (°C)
**M1-1K-TPU** ^1^	153,400	2.29	−12	77	1.6	123	3.4	314	368
**M2-1K-TPU**	152,300	2.64	0.7	77	2.5	127	4.2	306	346
**M3-1K-TPU**	73,600	2.79	−2.9	77	1.8	117	3.9	301	338
**M3-1.5K-TPU** ^2^	121,400	2.20	−16	79	0.5	132	6.4	299	342
**PTMG-1K-TPU**	95,400	2.68	−51	79	1.6	144	5.2	306	346, 423

^1^ *T*_m1_ and △*H*_m1_ of **M1-1K-TPU** are 20 °C and 6.6 J g^−1^, respectively; ^2^ content of hard segment is 38 wt % for TPUs containing macrodiols of 1 kg mol^−1^, except 29 wt % for **M3-1.5K-TPU**; ^3^ determined by THF-GPC using polystyrene standards (RI detector); ^4^ measured by DSC with a heating rate of 10 °C min^−1^ (1st scan); ^5^ measured by TGA with a heating rate of 10 °C min^−1^.

**Table 3:** Mechanical characterizations of TPU. UTS, ultimate tensile strength.

Sample	Young’s Modulus ^1^ (MPa)	Modulus at 100% Strain (MPa)	UTS (MPa)	Elongation at break (%)	Toughness ^2^ (MJ m^−3^)
**M1-1K-TPU**	14 (±0.3)	6.9 (±0.1)	56 (±4.2)	580 (±40)	140 (±20)
**M2-1K-TPU**	16 (±2.3)	14 (±1.4)	65 (±5.0)	440 (±8)	140 (±8)
**M3-1K-TPU**	12 (±0.5)	9.1 (±0.01)	46 (±2.4)	470 (±10)	100 (±2)
**M3-1.5K-TPU**	11 (±0.3)	5.7 (±0.02)	44 (±2.1)	680 (±18)	130 (±9)
**PTMG-1K-TPU**	6.5 (±1.3)	3.3 (±0.2)	9.9 (±2.9)	650 (±140)	40 (±17)

^1^ Measured by universal testing machine (UTM) with a pulling rate of 100 mm^−1^ at 25 °C; ^2^ Tensile toughness calculated by integrating the area under tensile curves.

## Supplementary Materials

The following are available online at www.mdpi.com/2073-4360/9/12/663/s1. Synthesis of carbonate-type macrodiols by Ti-catalyzed polycondensation of DPC and co-diols; Figure S1: DSC curves of macrodiols (second heating); Figure S2: Photograph of the carbonate-type macrodiol synthesized from diphenyl carbonate and titanium (IV) butoxide; Figure S3: Photographs of cross-linked gels when macrodiols without the neutralization were used for the TPU synthesis; Scheme S1: Plausible cross-linking reaction mechanism when remaining base chemicals activate bis-isocyanate-terminated pre-oligomers; Figure S4: ^1^H NMR spectrum of PTMG-1K-TPU in CDCl3 (300 MHz); Figure S5: GPC curves for synthesized TPUs; Figure S6: Optical microscopy images of the X-shaped scratch on TPU films.
