# Supplementary material for: Environmentally-Friendly Synthesis of Carbonate-Type Macrodiols and Preparation of Transparent Self-Healable Thermoplastic Polyurethanes
Source: Polymers (Basel). 2017 Nov 30;9(12):663. doi: 10.3390/polym9120663 (PMC6418697; doi:10.3390/polym9120663)
Supplement: Supplementary file 1 [file polymers-09-00663-s001.pdf]

Supplementary Materials: Environmentally-Friendly Synthesis of Carbonate-Type Macrodiols and Preparation of Transparent Self-Healable Thermoplastic Polyurethanes

Seon-Mi Kim, Seul-A Park, Sung Yeon Hwang, Eun Seon Kim, Jonggeon Jegal, Changgyu Im, Hyeonyeol Jeon, Dongyeop X. Oh and Jeyoung Park

Synthesis of carbonate-type macrodiols by Ti-catalyzed polycondensation of DPC and co-diols

A typical synthetic procedure of **M3-1K** (target M_n of 1 kg mol^{-1} from triple diol-monomers, molar feed ratio of HD:BD:PD = 4:4:2) is described below. HD (4.04 g, 68 mmol), BD (6.16 g, 68 mmol), PD (9.45 g, 34 mmol), DPC (31.5 g, 147 mmol) and titanium (IV) butoxide (0.0055 g, 250 ppm) were added to a 500-mL round-bottomed flask equipped with a distillation apparatus. The reaction temperature was increased to 140 °C and maintained for 2 h under a nitrogen atmosphere. The generated transesterification byproduct, phenol, was gradually distilled off. The distillation pressure was steadily reduced to 10 Torr for 6 h, while the temperature was gradually increased to 180 °C. After the pressure was returned to atmospheric pressure by a nitrogen purge followed by cooling to room temperature of 25 °C, the reactant dried under vacuum at 80 °C for 24 h. M_n (NMR): 900 g mol^{-1}, M_n (titr): 860 g mol^{-1}, ^{1}H NMR (300 MHz, $CDCl_3$): δ 4.28–4.03, 3.77–3.53, 1.88–1.74, 1.66–1.56, 1.54–1.49, 1.44–1.30, 1.01–0.89.

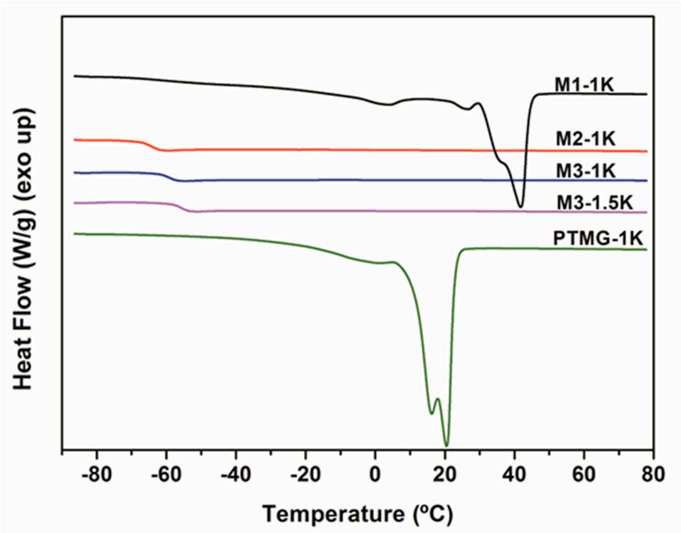

Figure S1. DSC curves of macrodiols (second heating).

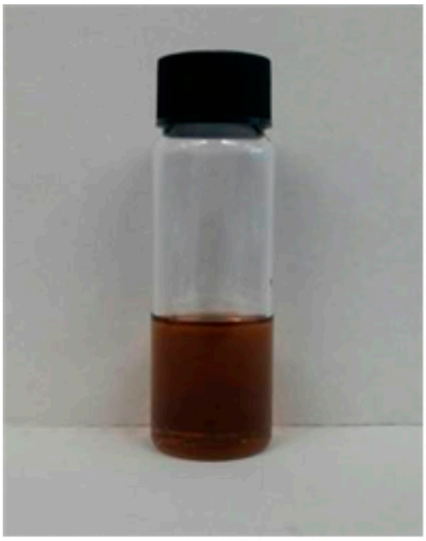

Figure S2. Photograph of the carbonate-type macrodiol synthesized from diphenyl carbonate and titanium (IV) butoxide.

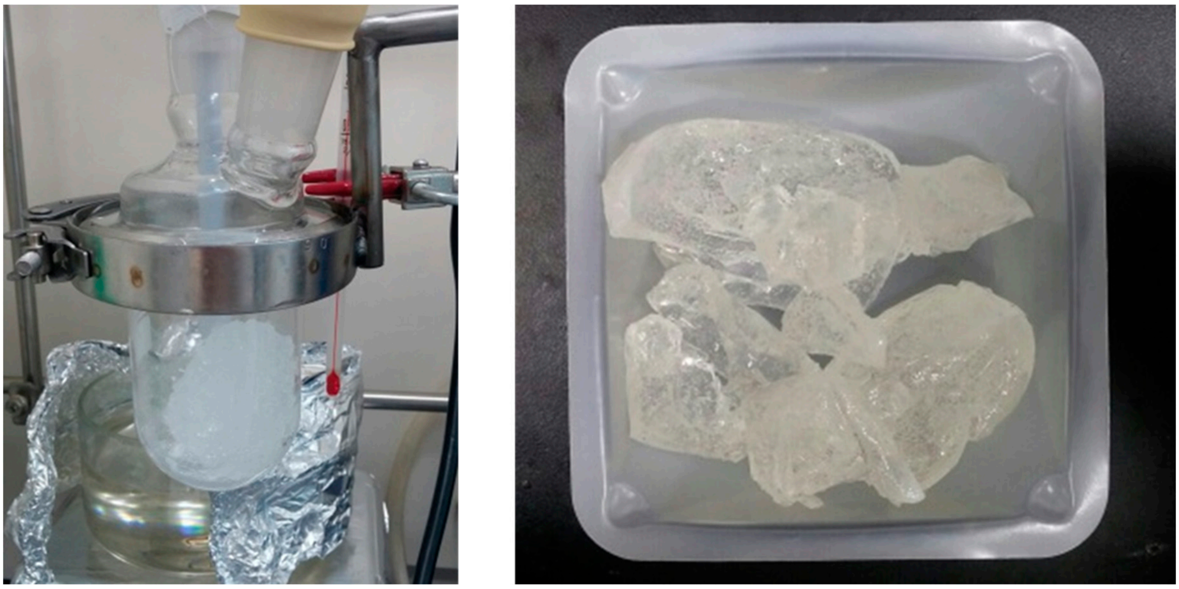

Figure S3. Photographs of cross-linked gels when macrodiols without the neutralization were used for the TPU synthesis.

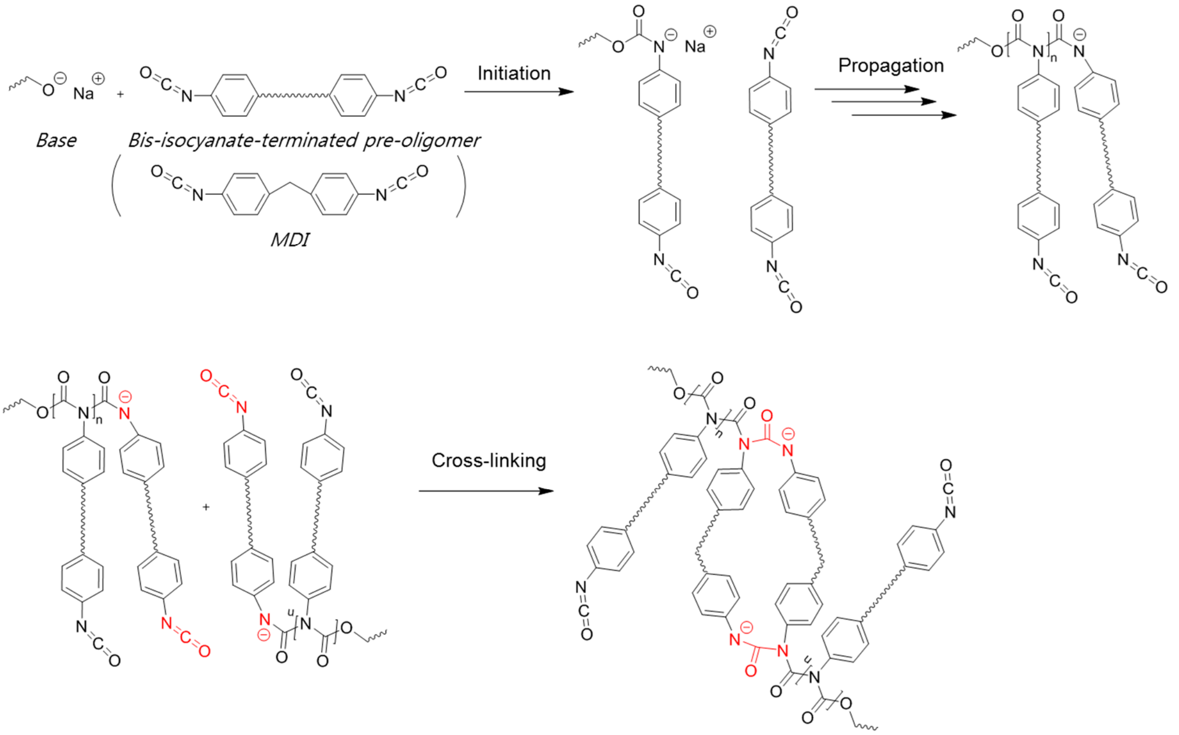

Scheme S1. Plausible cross-linking reaction mechanism when remaining base chemicals activate bis-isocyanate-terminated pre-oligomers. MDI, 4,4′-methylene(bisphenyl isocyanate).

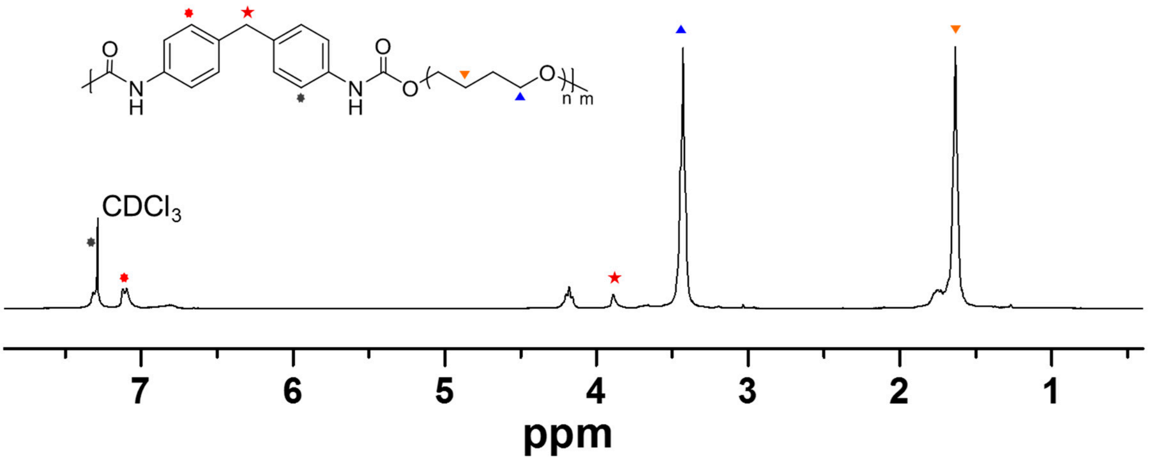

Figure S4. ^{1}H NMR spectrum of **PTMG-1K-TPU** in $CDCl_3$ (300 MHz).

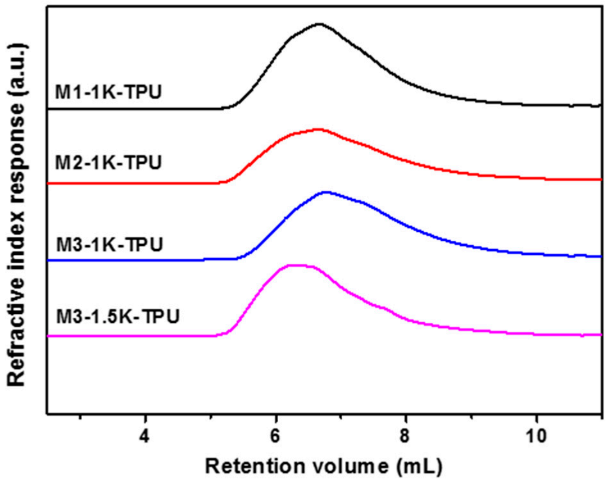

Figure S5. GPC curves for synthesized TPUs.

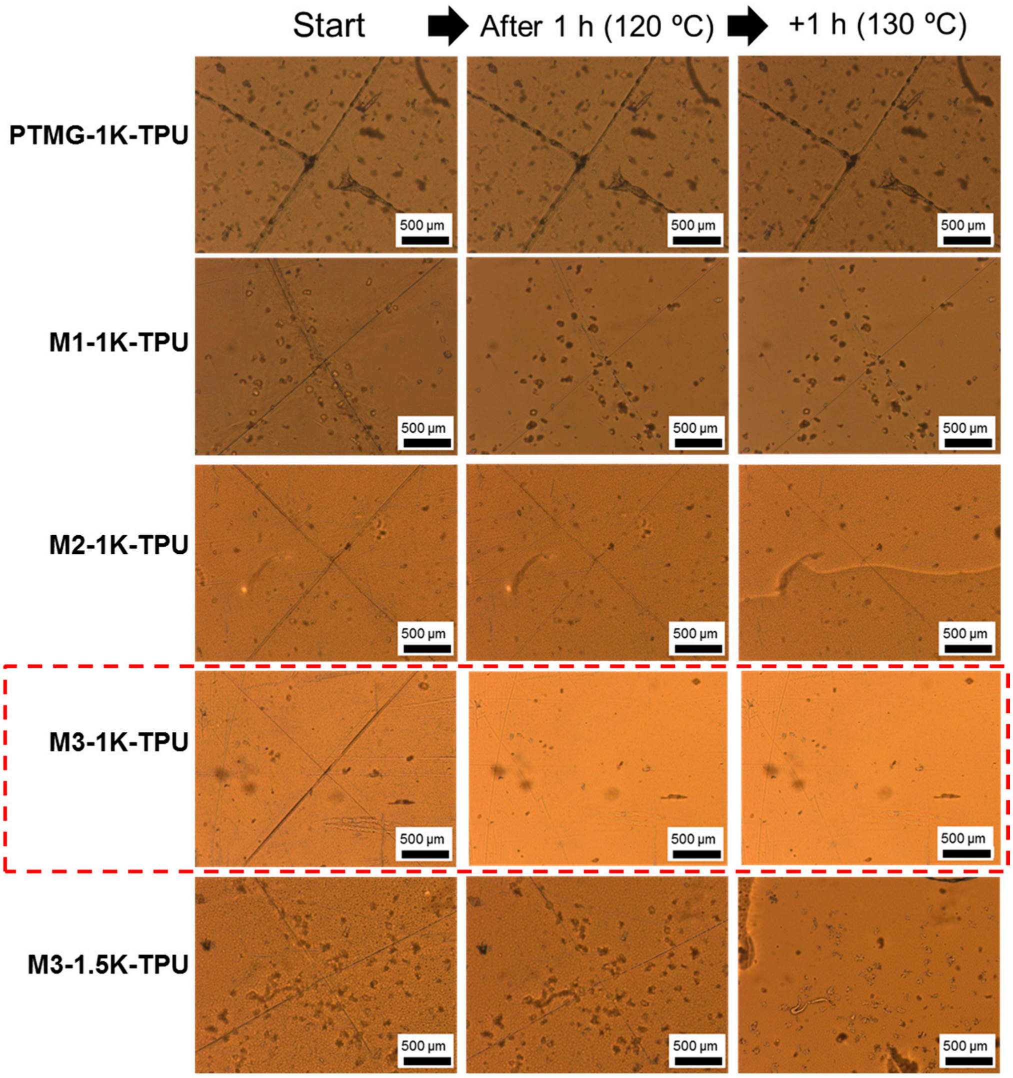

Figure S6. Optical microscopy images of the X-shaped scratch on TPU films.

© 2017 by the authors. Licensee MDPI, Basel, Switzerland. This article is an open access article distributed under the terms and conditions of the Creative Commons Attribution (CC BY) license (http://creativecommons.org/licenses/by/4.0/).
